# Submicron polymer particles may mask the presence of toxicants in wastewater effluents probed by reporter gene containing bacteria

**DOI:** 10.1038/s41598-021-86672-7

**Published:** 2021-04-01

**Authors:** Bhuvaneshwari Manivannan, Evgeni Eltzov, Mikhail Borisover

**Affiliations:** 1 Institute of Soil, Water and Environmental Sciences, Agricultural Research Organization, The Volcani Center, P.O. Box 15159, 7505101 Rishon LeZion, Israel; 2 Institute of Postharvest and Food Science, Department of Postharvest Science, Agricultural Research Organization, The Volcani Center, Rishon LeZion, Israel

**Keywords:** Ecology, Environmental sciences, Limnology, Natural hazards

## Abstract

Microplastics are ubiquitous in aquatic systems and break down into submicron particles that can interact with aquatic toxic chemicals. These interactions may affect the detection of toxicants when using bacteria as a biomonitoring tool. This study examined the effects of model polystyrene (PS)-based submicron particles on the detection of aqueous geno- and cytotoxicity by genetically modified bioluminescent (GMB) bacteria. The toxicities were tested in three treated wastewater (TWW) effluents before and after chlorination. The PS plastics included negatively charged sulfate-coated (S-PS) and pristine (P-PS) particles of different sizes (0.1, 0.5, and 1.0 µm) that were present at different concentrations. Chlorinated or not, the S-PS and P-PS particles per se were not toxic to the GMB bacteria. However, exposure of PS particles to TWW effluents can significantly reduce the measured geno- and cytotoxicity. Adsorption of toxic compounds to polymer particles can limit the ability of the bacteria to detect those compounds. This masking effect may be mitigated by TWW chlorination, possibly due to the formation of new toxic material. Due to interactions between toxic TWW constituents and the plastics particles, water samples containing particle-associated contaminants and/or their transformation products may be declared non-toxic, based on bacterial tests as a biomonitoring tool.

## Introduction

Microplastic particles of < 5 mm are ubiquitous in aquatic systems, where they have adverse effects on aquatic biota^1–3^. These microplastics can break down into nano-sized particles (~ 100 nm) through chemical and biological processes and become available for ingestion by zooplankton, fishes, bivalves, mussels, nematodes and crustaceans^4–9^. They may also be toxic to bacteria^10–13^. Plastic particles are capable of adsorbing multiple substances present in water, which affects their toxicity potential, as chemicals are concentrated and/or the particles become a vector for the spread of contamination^14–17^. The high surface area-to-volume ratio of small plastic particles may give those particles relatively large capacities for the adsorption of environmental contaminants present in aquatic systems^18,19^. Therefore, the presence of micro- and nano-sized polymer particles in water may affect the efficacy of biomonitoring tools to detect toxicological threats.

Bacteria are recognized as ecotoxicological bio-indicators for the evaluation of various environmental samples^20–23^. In particular, bacteria that exhibit reduced bioluminescence in the presence of various toxicants (such as *Vibrio fischeri*) or enhanced bioluminescence due to embedded reporter genes are well-known bioprobes for toxicity^24–28^. The latter approach using genetically modified bioluminescent (GMB) bacteria can provide rapid and sensitive luminescence-induction responses in the presence of toxic chemicals. It was used for examination of toxicity induced by atrazine^29^, 2,4,6-trinitrotoluene and 2,4-dinitrotoluene^30^, mitomycin C and phenol^31^, 2-chlorophenol and 4- nitrophenol^25^, butyl benzyl phthalate, 17β estradiol, paraquat, and hydrogen peroxide^32^. The GMB bacteria responses are also mechanism-specific, i.e., they may reflect cytotoxicity, genotoxicity or toxicity related to reactive oxygen species (ROS)^25–28,33^.

Little is known about how the interactions of micro- and nano-sized plastic particles with contaminants may interfere with the bacteria-based characterization of water toxicity induced by those contaminants^34^. Knowledge about such interfering interactions may be of particular significance for examining the toxicity of effluents released from wastewater treatment facilities and containing various plastic particles from industrial, agricultural and household systems^35–37^. Therefore, the general objective of this work was to characterize the effects of the interactions between polymer particles and contaminants present in treated wastewater (TWW) effluents on the toxicity of that TWW, as evaluated using GMB bacteria. Since wastewater treatment is often followed by disinfection, our interest also included the effects of contaminant‒polymer particle interactions on the GMB bacteria-probed toxicity of chlorinated TWW.

Polystyrene (PS)-based polymers were selected as the model plastic particles. They represent one of the most commonly produced polymers worldwide, with the highest abundance between 5 and 17% of total microplastics mass in wastewater effluents^35–37^, i.e., the third-highest among other polymer types. The wastewater may contain hundreds of microplastic particles of different types per liter, where after various treatments, their content may yet reach tens of particles per liter^38–41^. PS production is expected to reach about 10.6 million tons by the year 2023^42^. Also, the expanded PS containing 98% of air and developed as a low-density material for single-time uses is easily carried by winds and water and can increase the level of polymer contamination^43–45^. Multiple studies have examined the intrinsic toxicities of different PS particles toward bacteria^10,13,46^. PS has been shown to inhibit cell growth of the marine bacteria *Halomonas alkaliphila*^10^, control periphytic biofilm formation^47^ and increase antibacterial activity against *E. coli* due to exposure to UV radiation^46^.

On the other hand, Heinlaan et al.^12^ reported a non-toxic effect of 26-nm PS particles toward *E. coli*. Those particles did have a toxic effect on *V. fischeri*; the observed toxic effects were linked to surfactants and additives present in PS particles. Functional modifications of PS particles' surfaces may decrease the viability of *E. coli* cells^13^. Specifically, cationic amino-functionalized PS particles can lead to *E. coli* membrane destabilization and a ROS-mediated toxic effect^11^. These studies have proposed that the surface adhesion of PS-based particles to negatively charged bacterial cells is the primary cause of cell death in those systems. Therefore, surface-modified PS particles of different sizes and bearing negatively charged sulfate groups (S-PS) were used in this work. The negative charge leads to electrostatic repulsion between the bacteria cells and the plastic particles. Hence, direct interactions of the added PS particles with negatively charged bacterial cells could be weakened, thus allowing the focus on the toxicity induced by contaminants present in TWW. For comparison with negatively charged S-PS, one size of pristine PS was examined in the same systems. We expected that PS-based particles would remain chemically stable during the chlorination protocol applied^48–50^ and not produce any materials that would be toxic to the bacteria that we were using. Therefore, the hypothesis underlying this study was that due to adsorptive interactions of TWW components with an added polymeric material, the whole TWW toxicity probed with bacteria should decrease in the presence of submicron PS-based particles. This decrease will provide a quantitative estimate of the effect of plastic particles on the ability of GMB bacteria to detect toxic substances in TWW. In order to test this hypothesis and work toward the above-mentioned objective, the following experiments were performed: (1) testing the toxicity of the PS particles suspended in water toward GMB bacteria, prior to and after chlorination; (2) examining effects of added PS particles of different sizes, concentrations, and nature on the TWW toxicity and (3) comparing the toxicity of chlorinated TWW with that of TWW chlorinated in the presence of PS particles.

## Materials and methods

### Water sampling

Secondary TWW effluents were collected after primary filtration and activated sludge-based treatment, but before chlorination, from three municipal wastewater treatment plants situated in three Israeli cities: Rishon LeZion (i.e., Shafdan, Israel's largest wastewater treatment facility), Raanana and Karmi’el (referred to as S-TWW, R-TWW and K-TWW, respectively). The Shafdan wastewater treatment facility treats the daily flow of 360,000 m^3^ day^−1^ and serves approximately 2.3 million people in 35 municipalities^51^. The Raanana wastewater treatment plant treats 18,000 m^3^ day^−1^ of wastewater arriving from Raanana^52^ (with a population of about 75,000). The Karmi’el plant treats 36,500 m^3^ day^−1^ of wastewater arriving from 13 municipalities with a population of 210,000^53^. The effluents were filtered through a 0.45-µm filter (Whatman glass, Israel) and stored at 4 °C within a few hours after sampling. The filtered TWW effluent samples were characterized for pH, electrical conductivity (EC; Bante Instruments, DDs-12DW), and dissolved organic carbon (DOC) concentrations (TOC-L Rohs, Shimadzu; with the embedded solution acidification to eliminate carbonates). The pH values in the samples of S-TWW, R-TWW, and K-TWW were 7.4, 7.3, and 8.1, respectively. The EC values (µS cm^−1^) were 1105, 1110 and 1120 for S-TWW, R-TWW and K-TWW, respectively. The respective total concentrations of dissolved salts were 774, 778 and 795 mg L^−1^ in the same TWW series. The DOC concentrations (mg L^−1^) in S-TWW, R-TWW and K-TWW were 10.3, 8.2 and 12.9, respectively. From each wastewater treatment facility one TWW effluent sample was obtained. Hence, no characterization of wastewater treatment facilities may be derived from the data. Also, no relations or projections can be made towards seasonal, temporal or spatial variations of TWW effluent quality in treatment facilities. The only goal to use TWW samples from three essentially different treatment facilities separated geographically and serving different municipal areas was to examine whether adsorptive interactions of submicron plastic particles with contaminants present in TWW effluent samples may interfere with the bacteria-based characterization of water toxicity.

### Submicron polymer particles and preparation of solutions

The PS particles with negatively charged sulfate functional groups on their surface (tagged as S-PS) were obtained from Interfacial Dynamics Corporation (Thermo Fisher Scientific, USA) as milky aqueous suspensions. The S-PS particles used were of three sizes: 0.1 µm (8.1 g/100 mL), 0.5 µm (8.2 g/100 mL) and 1 µm (8.2 g/100 mL). The aqueous suspension of pristine 0.1-µm PS microspheres (referred to as P-PS; 10 g/100 mL) was purchased from Sigma Aldrich (Israel). For experimental studies, working PS concentrations of 20, 50 and 100 mg L^−1^ were prepared by diluting the stock solutions vortexed to minimize sedimentation. The high PS concentrations up to 100 mg L^−1^ were selected in order to clearly examine whether enhanced adsorptive interactions of TWW components with an added polymeric material make any impact on TWW effluent toxicity probed with bacteria. Yet, PS particles were considered to be relatively less toxic in this concentration range, following Heinlaan et al.^54^ reported that PS particles were toxic to most bacterial and algal model organisms at concentrations exceeding 100 mg L^−1^.

### Chlorination of TWW effluents and PS-particle suspensions

The sodium hypochlorite solution was used as a chlorinating agent. It was prepared from commercial NaOCl (CAS 425044, Sigma Aldrich) supposed to contain 12‒15% of available active chlorine. The precise hypochlorite concentration of the working stock solution (1000 mg L^−1^) was determined by measuring the absorbance of OCl^¯^ at 292 nm^55^. Absorbance measurements were made using a spectrophotometer (Thermo Scientific Genesys 10, Israel). Chlorination of the TWW effluents and the PS suspensions in TWW and Milli-Q water was performed in amber vials (3.0 mL) with minimal headspace at the chlorine concentration of 20 mg L^−1^ for 30 min, in the dark under shaking (100 rpm). This chlorination time was sufficient to inactivate coliform bacteria in water, at chlorine residual concentrations exceeding 0.7 mg L^−1^ ^55^. During the chlorination of the TWW effluents, the 20 mg L^−1^ concentration of chlorine was expected to generate significant concentrations of disinfection byproducts (DBPs) that can be sensed by GMB bacteria^26^. After the TWW chlorination, the residual chlorine concentration in each sample was quantified using *N,N*-diethyl-*p*-phenylenediamine (DPD) free-chlorine reagent (CAS 2105569, Hatch, USA). This DPD-based protocol is applicable for quantifying residual chlorine in various water samples including wastewater^56,57^. The quantification was performed by measuring spectrophotometrically the absorbance at 510 nm. In the presence of chlorine, the DPD amine is oxidized to Würster dye (magenta color) absorbing at 510 nm^56,58^. The standard curve was built with known chlorine concentrations of 0.05‒2 mg L^−1^; therefore, before determining the residual chlorine concentrations, the chlorinated TWW samples were diluted 10 times. To quench the residual chlorine, a solution containing stoichiometric amounts of sodium thiosulfate (CAS 72049, Sigma Aldrich) was used^56^.

When chlorinating suspensions of polymer particles in Milli-Q water, the residual chlorine concentration was taken just equal to the initial chlorine concentration of 20 mg L^−1^ and respectively quenched with the equivalent amount of sodium thiosulfate. Two tests supported this quenching approach. First, the 0.1-µm S-PS particles suspended at concentrations of up to 100 mg L^−1^ in Milli-Q water, either treated with 20 mg chlorine L^−1^ or not, did not produce any remarkable light-scattering or absorbance at 510 nm. When the 0.1-µm PS particles present at 100 mg L^−1^ in Milli-Q water were exposed to 20 mg L^−1^ of chlorine for 30 min, the residual chlorine concentrations were determined, using the DPD method, to be 19.5 ± 0.5 and 21.6 ± 2.1 mg L^−1^ for S-PS and P-PS particles, respectively. This result indicates that minimal reactions, if any, occurred between chlorine and the 0.1-µm PS particles under the conditions applied, and all of the applied chlorine remained active.

Second, the 0.5- and 1.0-µm S-PS particles in Milli-Q water at the 100 mg L^−1^ concentration were treated with 20 mg chlorine L^−1^. Then, these chlorinated suspensions were passed through a 0.22-µm filter (Whatman Puradisc 25 syringe filters, PTFE membrane). The PTFE is resistant to dry and wet chlorine and sodium hypochlorite solutions at up to 12‒14% mass concentration^59^. The residual chlorine concentrations in the filtered suspensions were determined using the DPD method and found to be 18.9 ± 1.4 and 19.0 ± 1.2 for the 0.5 and 1.0 µm S-PS particles, respectively. These results also indicate that the larger S-PS particles did not react substantially with chlorine. Based on the above-mentioned tests, residual chlorine concentrations after the chlorination of PS suspensions in Milli-Q water were considered similar to the initial chlorine concentration. Hence, they were quenched by the equivalent amount of sodium thiosulfate.

When chlorinating the TWW effluents containing polymer particles, the residual chlorine concentrations and the quenching amounts of sodium thiosulfate were also determined using the above-described DPD method, according to the absorbance measured at 510 nm; double-distilled water was used as a blank. Polymer particles in TWW cause light-scattering leading to apparently increased absorbance readings (except for 0.1-µm S-PS) and, hence, overestimating the amounts of residual chlorine determined using the DPD reagent (as above,^56,57^). Therefore, the amount of sodium thiosulfate used for quenching of chlorine excess was also overestimated, and it was present in some excess after the chlorination of TWW containing polymer particles.

### Characterization of toxicity using GMB bacteria

The geno- and cytotoxicity of the samples was evaluated with GMB bacteria obtained from Prof. Shimshon Belkin (Hebrew University of Jerusalem, Israel). The host *E. coli* DH5 alpha-RFM443 strains were genetically engineered to bear a bioluminescent reporter gene called *lux CDABE* in its plasmid, along with *recA* and *grpE* mechanism-specific promoters that respond to DNA damage and metabolic changes (heat-shock protein). These modified strains are referred to as DPD2794 (sensitive to genotoxicity) and TV1061 (sensitive to cytotoxicity)^25,60–63^.

Genotoxicity and cytotoxicity were measured before and after chlorination for (1) P-PS (0.1 µm) and S-PS (0.1, 0.5, 1 µm) particles suspended at concentrations of 20, 50 and 100 mg L^−1^ in Milli-Q water and (2) the TWW effluents (S-TWW, R-TWW and K-TWW) and TWW samples containing P-PS and S-PS particles at different concentrations. The chlorination step was always followed by quenching residual chlorine, as described in “Chlorination of TWW effluents and PS-particle suspensions” section, before any further toxicity measurements were made.

The protocol for assessing water toxicity using GMB bacteria was similar to that described previously^26,29^. In brief, the DPD2794 and TV1061 bacterial strains were cultured overnight in Difco Luria- Bertani (LB) Broth, Lennox, CAS 8011501) broth under shaking (125 rpm) at 37 °C in the presence of 50 µg L^−1^ ampicillin (CAS 69–52-3, Sigma Aldrich) to maintain the plasmids in the cells. The next day, 250 µl of culture was added to 25 ml of fresh LB broth and incubated for 5 h at 31 °C without shaking. Bacterial cells were centrifuged at 5000 rpm (Thermo Fisher Scientific) for 10 min at 4 °C, and pellets were re-suspended in fresh LB medium to 2.0 O.D. (optical density) and used for toxicity assays. The final sample volume of 200 µL contained a test sample and a bacterial cell culture at a 9:1 ratio (with a final O.D of 0.2). The samples prepared for the analysis were loaded into a 96-well white plate (SDL cell culture plate) for bioluminescence measurements under shaking. The bacterial luminescence was measured in relative light units by a luminometer (PerkinElmer, EnSpire 2300 Multilabel Reader, USA) at 420 nm and 26 °C. Measurements were taken every 5 min for 10 h. The bioluminescence ratio between a test sample and a control (Milli-Q water) was denoted as the induction factor (IF). An IF greater than 2 is considered to be a definite toxicity-induction pattern^64^.

Quenching of residual chlorine in TWW effluent samples containing polymer particles may be associated with overestimating the concentration of residual chlorine and the amount of sodium thiosulfate (“Chlorination of TWW effluents and PS-particle suspensions” section). The maximal apparent residual concentration of chlorine in TWW samples containing polymer particles did not exceed 15.2 mg L^−1^. To rule out the possible toxicity of excessive sodium thiosulfate, we tested the response of GMB bacteria to the concentration of sodium thiosulfate needed to quench 20 mg L^−1^ of chlorine. That response was below the threshold value (IF < 2). Toxicity appearing as general stress caused by salts in TWW effluents and reduced chemical potential of water was considered negligible. It is because the total dissolved-salt concentrations in the TWW effluents did not exceed 850 mg L^−1^ (as estimated using EC measurements). Our previous work^26,29^ demonstrated the lack of cytotoxic and genotoxic effects of saline solutions (with 1000 mg L^−1^ NaCl) on the GMB bacteria used in this study. However, specific toxic effects associated with the presence of certain inorganic and/or organic salts cannot be ruled out.

### Statistical analysis

All the experiments were carried out in triplicate, and the induction factors were expressed as means with standard errors. The statistical differences between the IF values were tested by unpaired multiple *t*-test comparisons according to the Sidak-Bonferroni method, using Graph Pad Prism statistical software (version 5). Statistical significance was defined as *p* < 0.05.

## Results and discussion

### Toxicity of PS-based particles in water, before and after chlorination

**Figure 1:**
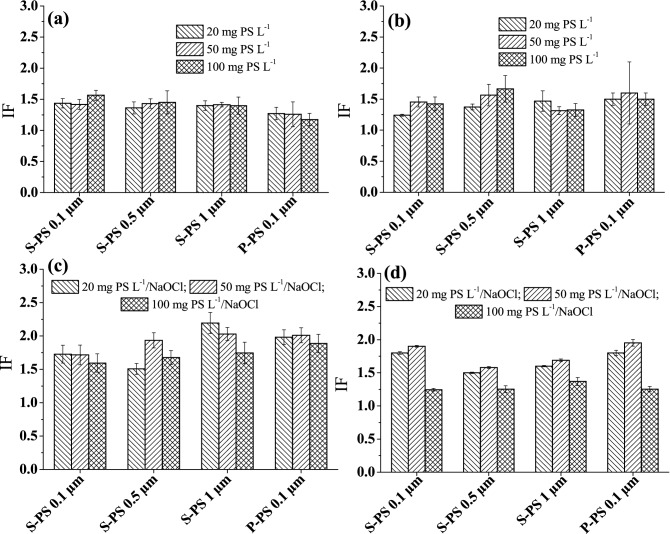
(**a**) Genotoxicity and (**b**) cytotoxicity of sulfate polystyrene (S-PS) particles of different sizes (0.1, 0.5 and 1 µm) and pristine, 0.1-µm polystyrene (P-PS) particles at particle concentrations of 20, 50 and 100 mg L^−1^ in Milli-Q water. (**c**) Genotoxicity and (**d**) cytotoxicity of S-PS and P-PS particles in Milli-Q water after chlorination (20 mg chlorine L^−1^). The error bars represent one standard deviation.

The geno- and cytotoxicity of the S-PS particles of different sizes (0.1, 0.5 and 1 µm) and the P-PS particles (0.1 µm) at particle concentrations of 20, 50 and 100 mg L^−1^ in Milli-Q water are shown in Fig. 1a,b. Irrespective of particle size, particle concentration and surface coating, IF values were less than 2, indicating that the PS particles per se did not induce significant bacterial bioluminescence and, therefore, were not toxic. The negative surface potential of sulfate PS particles^11^ could have hindered interactions with similarly charged bacterial cells^28^, as was expected when we selected our model polymer particles. P-PS particles have nearly non-charged, hydrophobic surfaces^65^. Nevertheless, interactions between cells and P-PS particles, if there were any, did not cause geno- or cytotoxicity that GMB bacteria could detect. It is also possible that the concentrations of PS particles used (≤ 100 mg L^−1^) were not sufficient to affect the bacterial strains. For example, Sun et al.^10^ showed that the exposure of the marine bacteria *Halomonas alkaliphila* to 55-nm plain PS particles produced a significant toxic effect only at a particle concentration exceeding 160 mg L^−1^. The formation of chlorine-carbon bonds during wastewater chlorination has been reported to increase the ecotoxicological effects of polymers^65^. However, the PS-based particles were selected in the expectation that they would remain chemically stable during the chlorination protocol applied^36–38^. Indeed, the chlorination of Milli-Q water containing S-PS and P-PS particles at different concentrations (20, 50 and 100 mg L^−1^) followed by the quenching of residual chlorine did not result in any cytotoxic or genotoxic effects (Fig. 1c, d). Generally, the IF values are less than or about 2, and they did not exceed 2 statistically significantly ( *p*< 0.05). This result suggests that the chlorination of PS particles did not produce any materials that were identified as toxic by the GMB bacteria. This finding is in agreement with the observation that the chlorine concentration did not decrease meaningfully in the presence of the PS particles suspended in Milli-Q water (Materials and Methods, “Chlorination of TWW effluents and PS-particle suspensions” section). Also, Kelker et al*.*^66^ showed that pristine PS particles are resistant to chlorination, based on minimal changes in Raman peak widths of PS sheets exposed to 75 mg min L^−1^ of chlorine.

### Genotoxicity of TWW effluents and the effects of PS particles

**Figure 2:**
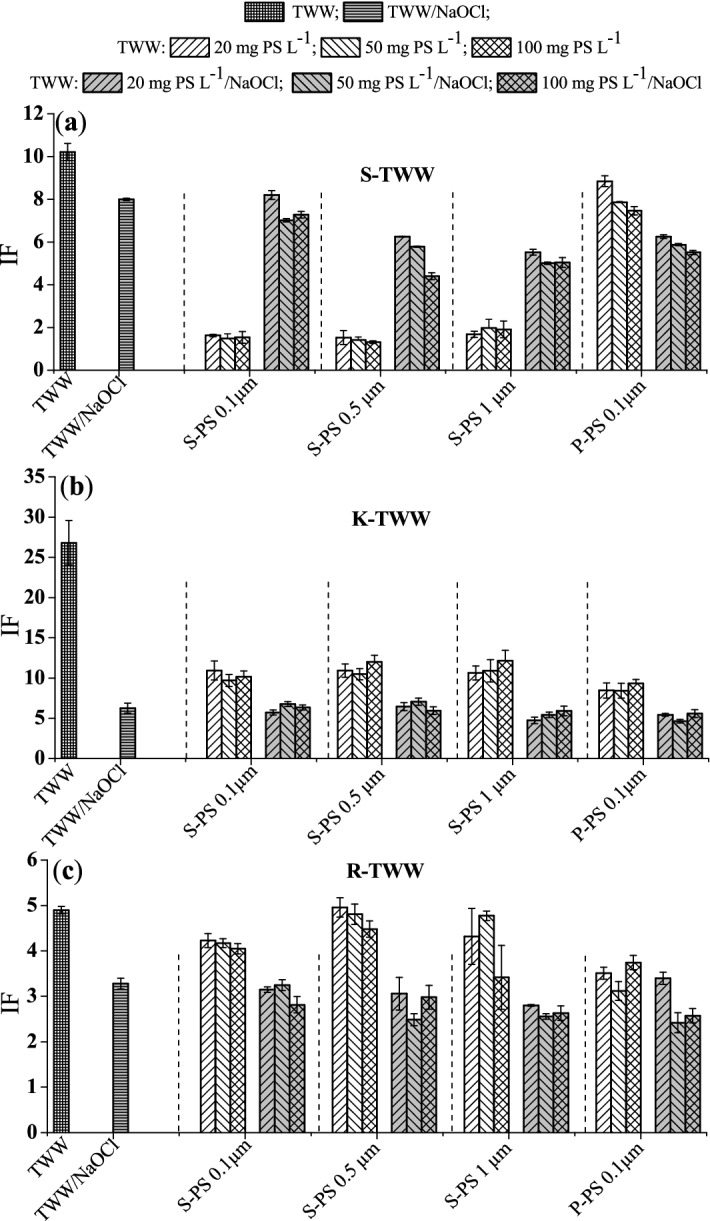
Genotoxicity of TWW effluent samples and the TWW-based suspensions of sulfate polystyrene (S-PS; 0.1, 0.5 and 1 µm) and pristine polystyrene (P-PS; 0.1 µm) particles at concentrations of 20, 50 and 100 mg L^−1^ before and after chlorination (20 mg L^−1^ of chlorine; by NaOCl). (**a**) Shafdan TWW effluent (S-TWW), (**b**) Karmi′el TWW effluent (K-TWW), (**c**) Raanana TWW effluent (R-TWW). The error bars represent one standard deviation.

To probe DNA damage and genotoxicity, we used the genetically engineered bacterial strain (DPD2794) bearing a reporter gene (*lux CDABE*) and the *recA* promoter. Induction factors (IF) quantifying the genotoxicity of the three TTWs studied, before and after chlorination, are presented in Fig. 2a‒c. The IF values of the non-chlorinated S-TWW, K-TWW and R-TWW samples were greater than 2 indicating their distinct genotoxicity. Upon exposure to S-PS particles of any size and concentration, the S-TWW effluent became non-genotoxic (IF < 2; Fig. 2a). This suggests that even the 20 mg L^−1^ concentration of S-PS particles was sufficient to mitigate the total genotoxicity potential of S-WW effluent, as detected using GMB bacteria. One explanation of that S-PS polymer particles' effect on genotoxicity is that submicron particles, having a high specific surface area, may effectively adsorb some toxic contaminants present in TWW. This adsorption decreasing the concentrations of toxic chemicals in the solutions would reduce their bioavailability. Figure 1 enforces this explanation by demonstrating that polymer particles are not toxic per se and cannot damage the bacteria. It should be kept in mind that the conclusion on non-toxicity of polymer particles is referred here to a specific PS polymer, either pristine or surface modified, of a specific shape (albeit of different sizes), and regarding the GMB bacteria used. Therefore, adsorbed chemicals are also unable to pass through bacterial cell membranes and produce damaging effects. In contrast to S-PS, the 0.1-µm P-PS particles, even at the highest examined concentration (100 mg L^−1^), contributed only to a small decrease in genotoxicity by the factor of 1.3, as compared to the IF of the source S-TWW effluent (Fig. 2a). This difference between S-PS and P-PS particles suggests that a significant portion of the S-TWW genotoxic components was positively charged. The positive charge makes them more effectively adsorbed on S-PS containing negatively charged sulfate groups, as compared with pristine PS particles. The lower adsorbing efficacy of P-PS particles is probably the reason for the statistically significant effect of P-PS particle concentration (with *p* < 0.05). The decrease in the genotoxicity of the non-chlorinated S-TWW induced by P-PS particles was enhanced as the particle concentration was increased (Fig. 2a).

Both the S-PS and the P-PS particles effectively suppressed, by 60‒68%, the genotoxicity of non-chlorinated K-TWW effluent (Fig. 2b), with no effect of particle size or concentration. P-PS was slightly more effective in suppressing the K-TWW genotoxicity than S-PS, which might be hypothesized to be caused by an effluent’s relatively high fraction of hydrophobic non-charged toxicants, as compared with S-TWW. These hydrophobic toxicants might adsorb effectively on hydrophobic P-PS surfaces. The genotoxicity of R-TWW effluent was minimal compared to that of S-TWW and K-TWW (Fig. 2c). The genotoxicity-reduction effect of added S-PS and P-PS particles was weak, in the 1.1‒1.3 range, if it existed at all, with slightly greater detoxification in the presence of P-PS as compared to S-PS (*p* < 0.05).

Chlorination of all three TWWs led to declines in genotoxicity detectable with the GMB bacteria, compared to genotoxicity of non-chlorinated TWWs (Fig. 2). This result is similar to the earlier observations^26^. It is also common that chlorination of wastewater produces DBPs, and leads to degradation of organics in water samples^26,66,67^. Thus, this decreased genotoxicity might reflect the combined effects of the degradation of some toxic materials and the formation of new chlorinated DBPs.

When S-TWW effluent was chlorinated in the presence of S-PS particles of different sizes at different concentrations (Fig. 2a), the genotoxicity generally decreased, as compared with S-TWW chlorinated in the absence of PS particles. However, this decrease did not exceed 45% and was much smaller than the > 80% decrease in the IF values caused by S-PS particles in the same non-chlorinated effluent. The weaker suppressing effect of PS particles on TWW toxicity after chlorination could be due to the formation of new genotoxic compounds in S-TWW during chlorination. The newly formed compounds could be adsorbed less effectively by PS particles and remain available for interactions with the GMB bacteria. These possibly oxidized substances may be hydrophilic and acidic. If such substances undergo dissociation, then, the produced anions would be poorly adsorbed by negatively charged S-PS particles. The genotoxicity IF values of S-TWW chlorinated in the presence of S-PS particles demonstrated certain irregular tendencies to decrease with increasing S-PS particle size and concentration (Fig. 2a). Increased adsorptive removal of some components at elevated particle concentrations might result in a decrease in genotoxicity.

The chlorination of S-TWW containing P-PS particles (Fig. 2a) led to genotoxicity IF values that were slightly lower than the IF values of non-chlorinated S-TWW containing P-PS particles (*p* < 0.05). This suggests that genotoxic substances present in S-TWW and not strongly adsorbed by P-PS are converted during chlorination into products with enhanced affinity to non-polar P-PS surfaces. The differences between the toxicity of S-TWW chlorinated in the presence of S-PS and P-PS particles and the toxicity of the same non-chlorinated systems might suggest that anionic hydrophobic substances are formed during chlorination (e.g., chloro-substituted ionizable phenols and/or carboxylic acids). The negatively charged substances are expectedly characterized by weakened adsorption to negatively charged surfaces (S-PS) but might demonstrate enhanced adsorption to the surface of non-polar P-PS particles.

When K-WW and R-WW effluents were chlorinated, the final genotoxicity was weakly affected or unaffected by the presence of S-PS or P-PS particles (Fig. 2b,c). In general, based on the data collected for the TWW effluents from three different sites, we can conclude that, when it persists, the PS-particle effect on the genotoxicity probed with GMB bacteria may be essentially reduced in chlorinated TWW effluent, as compared with the source TWW.

### Cytotoxicity of TWW effluents and the effects of PS particles

**Figure 3:**
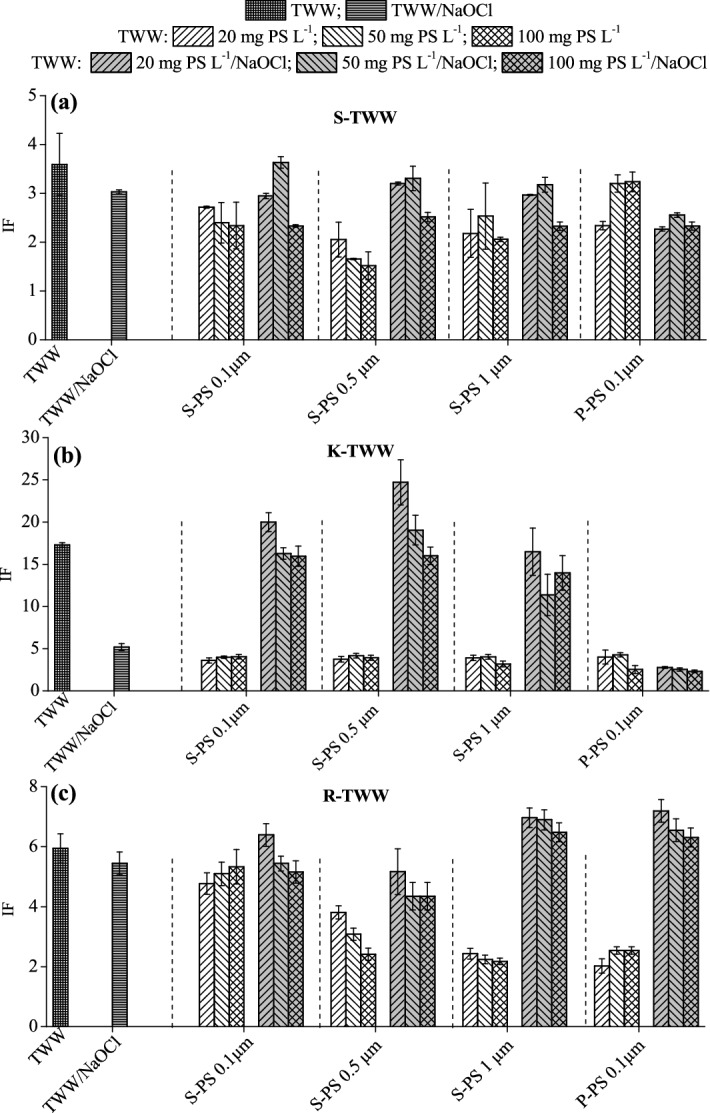
Cytotoxicity of TWW effluent samples and TWW-based suspensions of sulfate polystyrene (S-PS; 0.1, 0.5 and 1 µm) and pristine polystyrene (P-PS; 0.1 µm) particles at concentrations of 20, 50 and 100 mg L^−1^ before and after chlorination (20 mg L^−1^ of chlorine; by NaOCl). (**a**) Shafdan TWW effluent (S-TWW), (**b**) Karmi′el TWW effluent (K-TWW), (**c**) Raanana TWW effluent (R-TWW). S-TWW/NaOCl, R-TWW/NaOCl and K-TWW/NaOCl correspond to the chlorinated (20 mg L^−1^ of chlorine) TWW effluents. The error bars represent one standard deviation.

To probe cytotoxicity, we used a bacterial strain bearing *grpE* promoters and the *lux CDABE* reporter gene. Induction factors (IF) describing the cytotoxicity of TWWs, before and after chlorination, are presented in Fig. 3. The observed order of cytotoxicity (based on the IF values) was as follows: K-TWW > R-TWW > S-TWW. Compared to the source S-TWW effluent, the cytotoxicity of S-TWW effluent decreased distinctly in the presence of 0.5- µm and 1-µm S-PS (*p* < 0.05). The presence of 0.1-µm P-PS and S-PS particles resulted in smaller cytotoxicity decreases at the concentrations tested (Fig. 3a). Hardly meaningful effects of particle concentrations could be seen there. In the presence of S-PS and P-PS, there was a large decrease in the cytotoxicity of the K-TWW effluent (76‒85%; Fig. 3b). However, the effects of the type of PS particles, their size and concentration were not significant (*p* > 0.05). The addition of 1-µm S-PS and 0.1-µm P-PS particles to R-TWW effluent led apparently to significant and comparable removal of cytotoxic components, with no effect of particle concentration (Fig. 3c). The smaller S-PS particles (0.1 and 0.5 µm) had small to modest mitigating effects on the cytotoxicity of R-TWW.

The chlorination of S-TWW had no detectable effect on its cytotoxicity (Fig. 3a). In addition, chlorination in the presence of S-PS particles, except the 100 mg L^−1^ particle concentration, had hardly any effect on S-TWW cytotoxicity when compared with the S-TWW chlorinated without added S-PS. The high (100 mg L^−1^) concentration of S-PS tended to suppress the residual cytotoxicity of this TWW effluent during chlorination. In the result, its cytotoxicity became comparable to that measured in the S-TWW chlorinated at the presence of P-PS.

The chlorination of K-TWW effluent caused a distinct 70% decrease in its cytotoxicity (Fig. 3b). Interestingly, the chlorination of K-TWW effluent in the presence of S-PS particles caused a significant increase (119‒376%) in its cytotoxicity, as compared with that of the chlorinated source effluent. Since adding PS particles and chlorination alone strongly reduced the K-TWW cytotoxicity, the high cytotoxicity scores of the K-TWW chlorinated in the presence of PS particles may suggest that there was some formation of new cytotoxic DBPs. These DBPs were not present in the K-TWW chlorinated in the absence of plastic particles. The cytotoxicity of K-TWW chlorinated in the presence of S-PS particles was weakly, if at all, dependent on particle concentration, suggesting the weak interactions between the DBPs formed and the surfaces of the anionic S-PS. Chlorination of K-TWW containing P-PS particles resulted in cytotoxicity similar to that observed for the control (Fig. 3b). It was even less than that obtained after chlorinating the source K-TWW or after its interactions with virtually all types of PS particles without chlorination (at *p* < 0.05). Thus, the combined effects of chlorination and interactions with P-PS particle surfaces lacking negatively charged functional groups eliminated the cytotoxicity of the K-TWW effluent sample studied.

Chlorination did not alter the cytotoxicity of the source R-TWW effluent (Fig. 3c). Chlorination of R-TWW in the presence of 0.1-µm and 0.5-µm S-PS particles had no or marginal effects on cytotoxicity, compared to the R-TWW chlorinated alone. However, when R-TWW was chlorinated in the presence of large, 1.0-µm S-PS particles or 0.1-µm P-PS particles, its cytotoxicity exceeded that of both R-TWW chlorinated alone (by 19‒28% for 1.0-µm S-PS particles and 16‒32% for 0.1-µm P-PS particles) and that of R-TWW in contact with these plastic particles (by 190‒210% for 1.0-µm S-PS particles and 150‒260% for 0.1-µm P-PS particles). This elevated cytotoxicity suggests the formation of new toxic DBPs that were not present in the R-TWW chlorinated in the absence of plastic particles (as also suggested above regarding the K-TWW effluent chlorinated in the presence S-PS particles; Fig. 3b).

## Summary and conclusions

Pristine and sulfate-coated PS particles were not toxic to the GMB bacteria per se or after they were present during a chlorination procedure. However, they had variable effects on the geno- and cytotoxicity of the source TWW effluents and their chlorinated products. These effects might be differently and not always regularly influenced by the type, size or concentration of the PS particles and the nature of the effluents. Nevertheless, the data suggest that the adsorption of some water components to submicron polymer particles may make them "undetectable" by probing with bacteria. The adsorption of pollutants onto polymer particles is to be expected, based on multiple studies in the literature. This adsorption should depend on the nature of the plastic particles, their specific surface area, the coating functional groups and the chemical structure of the toxic substances. As an example, interactions between polymer particles coated with negatively charged groups and positively charged toxic contaminants may lead to an apparent decrease in sample toxicity monitored using bacteria, as proposed here based on the data shown in Figs. 2 and 3. Since the fate of toxic contaminants adsorbed by polymer particles is generally unknown, a given water sample may be declared non-toxic, overlooking potential desorption of plastic-associated contaminants and/or their transformation into even more toxic products capable of desorption. Thus, submicron polymer particles (represented in this work by PS) may mask the presence of toxic substances in TWW effluents and complicate the detection of those chemicals by bacteria.

It has been proposed in the literature that adsorption of contaminants to aged polymer particles is even more substantial than to fresh polymer surfaces^68^. Therefore, even more substantial masking effects might be expected in real environments. This masking effect of adsorbing polymer particles, observed in TWW effluent samples, may be weakened when water samples are chlorinated, as was seen from the genotoxicity and cytotoxicity data presented here. The mitigation of the plastic-particle effect could be caused by the formation of new DBPs during chlorination (e.g., hydrophilic and negatively charged DBPs that are less easily adsorbed onto negatively charged polymeric surfaces). Nevertheless, the provided data raise a concern regarding interference between the interactions of toxic TWW constituents with polymer particles and the detection of toxicological threats using bacterial probes.
